# Residue Elimination Patterns and Determination of the Withdrawal Times of Seven Antibiotics in Taihang Chickens

**DOI:** 10.3390/ani15152219

**Published:** 2025-07-28

**Authors:** Huan Chen, Cheng Zhang, Nana Gao, Guohua Yan, Yandong Li, Xuejing Wang, Liyong Wu, Heping Bai, Hongyu Ge, Huage Liu, Juxiang Liu

**Affiliations:** 1 College of Veterinary Medicine, Hebei Agricultural University, Baoding 071000, China; chenhuantx@163.com (H.C.); zc1349@foxmail.com (C.Z.); gaonanahbnd2023@163.com (N.G.); baiheping2020@163.com (H.B.); 2 Institute of Animal Husbandry and Veterinary Medicine of Hebei Province, Baoding 070066, China; yanguohua2022@126.com (G.Y.); wangxuejing771226@163.com (X.W.); 3 Hebei Provincial Station of Veterinary Drug and Feed, Shijiazhuang 050035, China; 15613108352@163.com (Y.L.); wuliyong@outlook.com (L.W.); 4 School of Life Sciences and Food Engineering, Hebei University of Engineering, Handan 056038, China; 18232120207@163.com

**Keywords:** antibiotic, Taihang chickens, residue elimination, WDT

## Abstract

Antibiotic residues in poultry pose health and resistance risks, necessitating breed-specific WDTs. In this study, the residue elimination patterns of seven antibiotics in Taihang chicken tissues under free-range conditions were studied and the appropriate WDT was formulated. A total of 240 healthy Taihang chickens aged 100 days were randomly divided into 8 groups, each comprising 30 chickens. Chickens in groups 1 to 7 were administered oxytetracycline, chlortetracycline, erythromycin, tylosin, tylvalosin, lincomycin, and tiamulin, respectively. Regarding the administration method, we adopted the highest dose and maximum course of treatment recommended by the Veterinary Pharmacopoeia of the People’s Republic of China. Group 8 served as the control group. Muscle, sebum, liver, and kidney samples were collected at 4 h, 1 d, 2 d, 3 d, 5 d, 7 d, 10 d, 13 d, and 16 d after drug withdrawal. Our results demonstrated that the drug residues after drug withdrawal gradually decreased with the increase in drug withdrawal days, and the elimination rate in the early stage of drug withdrawal was significantly faster than that in the later stage. At 4 h after drug withdrawal, the drug residues in various tissues reached their highest values. In most cases, the drug concentrations in the kidney and liver were higher than those in the muscles and sebum; however, some drugs also exhibited concentration peaks in the sebum. On the first day of drug withdrawal, the amount of residues in various tissues decreased rapidly. In general, the elimination rate of various drugs in the muscles, liver, and kidneys is faster but slower in the sebum. Based on the WDT calculation software WT1.4, the recommended WDTs for oxytetracycline, chlortetracycline, erythromycin, tylosin, tylvalosin, lincomycin, and tiamulin chickens are 4 d, 5 d, 11 d, 8 d, 13 d, 13 d, and 7 d, respectively. These findings support food safety and industry development.

## Simple Summary

Antibiotic residues in poultry not only present a threat to human health but also contribute to the development and spread of antibiotic resistance. Determining the withdrawal times (WDTs) for antibiotic use in broilers is therefore vital in this regard; however, to date, no reports specifically addressing WDTs in Taihang chickens have been published. The objective of this study was to investigate the residue elimination patterns of seven antibiotics in free-range Taihang chickens and establish appropriate WDTs. The results demonstrated that the recommended WDTs for oxytetracycline, chlortetracycline, erythromycin, tylosin, tylvalosin, lincomycin, and tiamulin in Taihang chickens are 4 days, 5 days, 11 days, 8 days, 13 days, 13 days, and 7 days, respectively. These findings provide data support for establishing WDTs of antimicrobial agents in Taihang chickens to ensure rational drug use and food safety and offer technical references to safeguard the development of the Taihang chicken industry.

## 1. Introduction

With the substantial improvement in the Chinese economy and people’s living standards, the proportion of animal-derived foods in the daily dietary consumption of urban and rural residents has increased year-on-year. China has become the world’s largest producer of animal-derived foods and stands as the country with the highest total consumption [1]. Poultry, as a major pillar of animal-derived foods, accounts for approximately 36% of global meat production [2]. The poultry industry has developed into one of the largest livestock sectors worldwide, and antibiotics are widely used in poultry production [3]. Antibiotic residues in poultry pose a threat to human health (including teratogenic, carcinogenic, and mutagenic effects), particularly in muscle, sebum, liver, and kidney tissues. These residues induce bacterial resistance, leading to treatment failure in humans [4,5]. In 2019, China published the national food safety standard GB 31650-2019 “National Food Safety Standard—Maximum Residue Limits for Veterinary Drugs in Foods” [6]. Based on this standard, the maximum residue limits (MRLs) for oxytetracycline and chlortetracycline in chicken tissues are 200 μg/kg in muscles, 600 μg/kg in the liver, and 1200 μg/kg in the kidney. Regarding erythromycin and tylosin, the MRLs in chicken tissues (muscle, sebum, liver, and kidney) are uniformly 100 μg/kg. The MRL of tylvalosin is 50 μg/kg in the sebum, liver, and kidney. The MRLs of lincomycin are 200 μg/kg in muscles, 100 μg/kg in fat, and 500 μg/kg in the liver and kidney, respectively. The MRLs of tiamulin are 100 μg/kg in muscles and sebum and 1000 μg/kg in the liver. The residue markers for oxytetracycline, chlortetracycline, lincomycin, and tiamulin in tissues are the parent compounds themselves. Regarding erythromycin and tylosin, their residue markers are erythromycin A and tylosin A, respectively; tylvalosin’s residue markers include both tylvalosin and 3-O-acetyltylosin.

Taihang chickens, originally referred to as “Hebei Chai chickens”, are a unique local breed in China, primarily distributed in Hebei Province and renowned for their high-quality meat and eggs [7,8]. Taihang chicken meat is characterized by its rich aroma, tender texture, and distinctive flavor, with the eggs of the chickens exhibiting superior quality and nutritional value. The broth prepared from Taihang chickens possesses an exceptionally rich flavor, making it incredibly popular among consumers [9,10]. However, limited data exist on the residue elimination patterns of the seven aforementioned antibiotics in Taihang chickens. The aim of the present study was to therefore investigate the antibiotic residue elimination profiles across various tissues of Taihang chickens.

To ensure the food safety of Taihang chicken products, the authors of previous studies have focused on the residue elimination of seven antibiotics in eggs produced by this type of chicken [11], establishing WDTs specifically for eggs. However, the determination of WDTs requires the comprehensive consideration of drug residue patterns and elimination dynamics in the muscle, skin and fat, liver, kidney, and other tissues [12,13]. By monitoring and analyzing these tissues and organs, scientifically rational WDTs could be established to ensure the food safety of Taihang chicken products and protect consumer health.

## 2. Materials and Methods

### 2.1. Animals

The Taihang chickens used in this study were purchased from Shijiazhuang Zanhuang Natural Agricultural Products Co., Ltd., Shijiazhuang, China (at the age of 100 days; initial body weight: hens 840 ± 15 g and roosters 1189 ± 22 g) [14]. A total of 240 healthy Taihang chickens were randomly divided into 8 groups, each comprising 30 chickens (6 chickens per cage, 5 replicates). Chickens in groups 1 to 7 were administered oxytetracycline, chlortetracycline, erythromycin, tylosin, tylvalosin, lincomycin, and tiamulin, respectively. Group 8 served as the control group. Before the experiment, the 6 animals in each cage were numbered. They were in good mental health condition. One week before the start of the experiment, all Taihang chickens were uniformly managed. During the experiment, all groups of Taihang chickens were kept under similar management conditions and in a controlled microclimate (temperature: 21–22 °C; humidity: 50–60%). The lighting duration was 16 h (from 06:00 to 22:00), and nipple drinkers were used to feed the chickens. During the adaptation period, commercial feed without antibiotics and access to water were provided to the chickens ad libitum. The basal diet consisted of corn–soybean meal, which is a commercial grain from Tianniu Company (Shijiazhuang, China). The health status of the chickens (including water intake and feed intake) was observed every day. This study complied with standard ethical requirements, and the animal experiment protocol was approved by the Animal Ethics Committee of Hebei Agricultural University (approval number: 20241426; approval date: 23 May 2024).

### 2.2. Drugs and Reagents

The manufacturers, batch numbers of drugs and the WDTs specified in the Chinese Veterinary Pharmacopoeia are shown in Table 1.

### 2.3. Experimental Design

**Figure 1:**
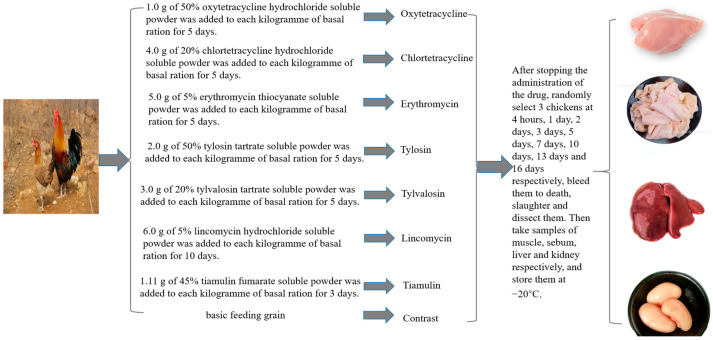
Experimental design for antibiotic administration and tissue sampling in Taihang chickens.

In accordance with the “Guidelines for Veterinary Drug Residue Elimination Test” No. 326 of the Ministry of Agriculture and Rural Affairs (2020), the experimental group was administered the maximum dose and longest course of treatment recommended by the veterinary pharmacopoeia, while the control group was not treated with any antimicrobial drugs. The specific experimental grouping methods and dosages are shown in Figure 1.

### 2.4. Sample Preparation

All samples were first thawed to room temperature. The muscle, skin and fat, liver, and kidney tissues were homogenized using a GM200 high-speed dispersing homogenizer (Retsch GmbH, Haan, Germany). A previously described protocol was implemented for the extraction of drugs from muscle, skin and fat, liver, and kidney samples [11,15] with minor modifications. Accurately weighed 2.0 g of tissue homogenate was transferred into a 50 mL polypropylene centrifuge tube. Subsequently, 2 mL of acidified water (0.2% formic acid) was added, and the mixture was vortexed for 10 min. Thereafter, 8 mL of acidified acetonitrile (0.2% formic acid) was added, followed by vortexing for 10 min and ultrasonication for 10 min. The mixture was then centrifuged at 12,000 rpm for 10 min. The Oasis PRIME HLB solid-phase extraction (SPE) cartridges (Waters, Milford, MA, USA) were used without prior activation or equilibration. The supernatant was purified through the SPE cartridges at a flow rate of one drop per second, and 3 mL of the filtrate was collected. The 3 mL supernatant was evaporated to dryness under a nitrogen stream at 40 °C. The residue was reconstituted in 0.6 mL of mobile phase (0.1% formic acid in water–acetonitrile (96:4, *v*/*v*)) and filtered through a 0.22 μm GHP membrane filter for LC-MS/MS analysis.

### 2.5. Liquid Chromatography–Mass Spectrometry Conditions

The ultra-performance liquid chromatography–tandem mass spectrometry (UPLC-MS/MS) system used in this study was a Waters Acquity UPLC Xevo TQ-S (Waters, USA). The chromatographic conditions were as follows: The analytical column was a C18 column (HSS T3, 50 mm × 2.1 mm inner diameter, 1.8 μm particle size). The mobile phase consisted of 0.1% formic acid in water (A) and acetonitrile (B). A gradient elution program was applied as follows: 0~2.5 min: 98% A, 2% B; 2.5~4 min: 80% A, 20% B; 4~4.5 min: 10% A, 90% B; 4.5~5 min: 98% A, 2% B. The flow rate was 0.4 mL/min, the column temperature was maintained at 30 °C, and the injection volume was 2 μL.

The mass spectrometric conditions were as follows: The electrospray ionization (ESI) source was operated in positive ion mode. The scanning mode was dynamic multiple reaction monitoring (MRM). Key parameters for the ESI + ion source were set as follows: capillary voltage: 3.00 kV; desolvation temperature: 400 °C; cone voltage: 65 V; desolvation gas flow: 850 L/h; collision gas flow: 0.15 mL/min. Characteristic ions were based on the mass spectrometry conditions listed in Table 2.

Tissue samples from the control group (muscle, skin and fat, liver, and kidney) were collected as blank samples. The matrix solution of blank tissues was prepared following the procedures described in Section 2.4. The standard working solutions were serially diluted to prepare matrix-matched standard solutions at concentrations of 0.5, 1.0, 5.0, 20, 50, 100, and 200 ng/mL using the blank matrix. These solutions were then analyzed via ultra-performance liquid chromatography–triple quadrupole tandem mass spectrometry (UPLC-MS/MS).

Spiked blank samples were prepared by adding the seven analytes at three concentrations (5, 10, and 50 μg/kg) to blank tissue matrices. For each concentration level, five replicate samples were prepared, and three independent batches were processed. The recovery rates, intra-day recovery, inter-day recovery, and coefficients of variation (CV) were calculated to validate the method’s accuracy and precision.

### 2.6. Method Validation

The analytical method was comprehensively validated in accordance with the European Commission Directive 2002/657/EC. A series of matrix-fortified solutions containing the seven analytes at varying concentrations were used to construct calibration curves. The results demonstrated that all seven analytes exhibited good linearity within the range of 1.0 to 200 ng/mL. Blank tissue matrices (muscle, skin and fat, liver, and kidney) spiked with appropriate amounts of the seven antibiotic standard working solutions were quantified using the calibration curves. By analyzing these spiked blank samples, the limits of detection (LOD) and limits of quantification (LOQ) were determined as the lowest analyte concentrations yielding chromatographic peaks at signal-to-noise ratios of 3 (LOD) and 10 (LOQ), respectively.

The decision limit (CCα) and detection capability (CCβ) were calculated using the method described by Verdon et al. [16]. Accuracy and precision were evaluated by analyzing the spiked blank samples at three concentration levels over three days, with six independent replicates prepared for each concentration level.

## 3. Results

### 3.1. Method Validation Results

The UPLC-MS/MS method employed in this study demonstrated specificity for muscle, skin and fat, liver, and kidney tissues, with no interfering peaks observed at the retention times of the seven antibiotics. Good linearity was achieved within the range of 1.0–200 ng/mL, with correlation coefficients (R^2^) exceeding 0.995. For samples exceeding the upper concentration limit, appropriate dilution in the mobile phase was performed, followed by re-quantification, as described earlier. The limits of quantification (LOQ) and limits of detection (LOD) were defined as the analyte concentrations corresponding to signal-to-noise (S/N) ratios of ≥10 and ≥3, respectively. The regression equations, correlation coefficients, LOD, and LOQ are summarized in Table 3.

**Figure 2:**
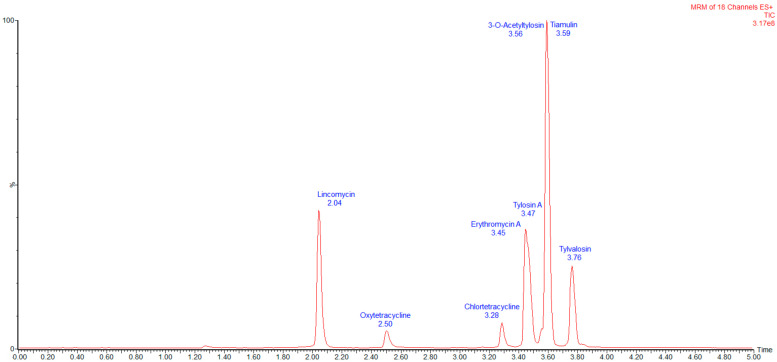
Total ion current chromatogram of the seven types of antibiotics in muscle, skin and fat, liver, and kidney samples spiked at 50 μg/kg. Note: Erythromycin A and tylosin A retention times are similar and overlap in the total ion flow diagram. 3-O-Acetyltylosin and tiamulin retention times are similar and overlap in the total ion flow diagram. The quantitative ionogram is shown in Figure 3.

To facilitate validation, blank muscle, skin and fat, liver, and kidney samples were spiked with mixed standard working solutions at the LOQ, 2LOQ, and 5LOQ levels. Six replicates were prepared for each concentration across three independent batches. The recovery rates ranged from 71% to 99%, with intra-batch and inter-batch precision (expressed as relative standard deviation (RSD)) of ≤4.4%. Detailed recovery rates, precision values, CCα, and CCβ are provided in Table 4. The total ion chromatograms (TICs) of the seven antibiotics in spiked muscle, skin and fat, liver, and kidney samples are shown in Figure 2.

**Figure 3:**
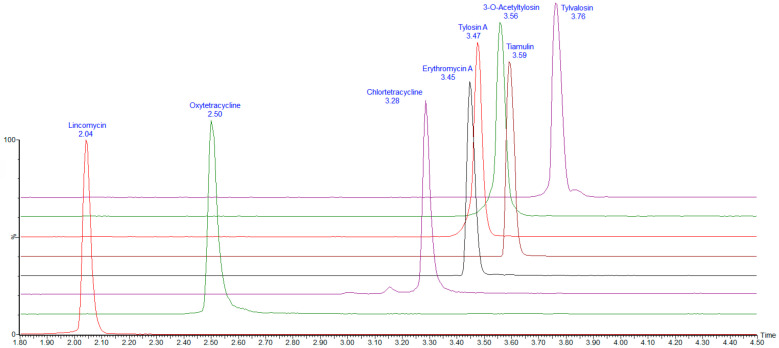
Quantitative ionogram of muscle, skin and fat, liver, and kidney matrices spiked with seven antibiotic standards at 50 μg/kg.

### 3.2. Residue Elimination Pattern

Throughout the entire experimental process, all Taihang chickens demonstrated robust health conditions without any obvious adverse reactions, and their water and food intake were recorded. During dissection, all tissues and organs were found to be in good condition with no lesions.

#### 3.2.1. Elimination Pattern of Oxytetracycline and Chlortetracycline, and Their Main Metabolites

As shown in Figure S1, residues were detected in different tissues, such as those of the muscles, skin and fat, liver, and kidney, indicating that oxytetracycline and chlortetracycline can be widely distributed after entering the body. Four hours after drug withdrawal, the residue levels of oxytetracycline and chlortetracycline reached their peak in the muscles, skin and fat, liver, and kidney, and the concentration was the highest in the kidney. On the first day after drug withdrawal, the concentrations of oxytetracycline and chlortetracycline remaining in various tissues decreased rapidly. On the first day after drug withdrawal, the levels of oxytetracycline in the muscles, liver, and kidney all dropped below the residue limits stipulated in GB 31650-2019 (200 μg/kg, 600 μg/kg, and 1200 μg/kg, respectively). Four hours after drug withdrawal, the levels of chlortetracycline in the muscles and liver dropped below the residue limits stipulated in GB 31650-2019 (200 μg/kg and 600 μg/kg, respectively), and the level of chlortetracycline in the kidney was 1320 μg/kg at this time, with it dropping below the limit (1200 μg/kg) only on the first day after drug withdrawal. During the drug withdrawal period, the contents of oxytetracycline and chlortetracycline in the skin and fat, muscles, liver, and kidney generally decreased with the increase in the number of days of drug withdrawal. The elimination speed was faster in the early stage of drug withdrawal than in the later stage. Oxytetracycline and chlortetracycline were eliminated rapidly in the liver, kidney, and muscles but slowly in the skin and fat.

#### 3.2.2. Elimination Pattern of Erythromycin, Tylosin, and Tylvalosin, and Their Main Metabolites

As shown in Figure S2, four hours after drug withdrawal, the sum of erythromycin A, tylosin A, tylvalosin, and 3-O-acetyltylosin reached the highest residue levels in the muscles, skin and fat, liver, and kidney. Erythromycin A reached the highest concentration in the kidney, while the sum of tylosin A, tylvalosin, and 3-O-acetyltylosin reached the highest concentration in the skin and fat. On the first and second days after drug withdrawal, the concentrations of the sum of erythromycin A, tylosin A, tylvalosin, and 3-O-acetyltylosin remaining in the various tissues decreased rapidly. The period when erythromycin A dropped below the residue limits stipulated in GB 31650-2019 in the muscles, skin and fat, liver, and kidney was 4 h after drug withdrawal, the 3rd day after drug withdrawal, and the 1st day after drug withdrawal, respectively. Four hours after drug withdrawal, the residues of tylosin A in the muscles, liver, and kidney dropped below the residue limit stipulated in GB 31650-2019 (100 μg/kg). Tylosin A in the skin and fat could only drop below the limit (100 μg/kg) on the third day after drug withdrawal. The periods when the sum of tylvalosin and 3-O-acetyltylosin dropped below the residue limits stipulated in GB 31650-2019 (50 μg/kg) in the skin and fat and liver were the fifth day and second day after drug withdrawal, respectively. The elimination speed was faster in the early stage of drug withdrawal than in the later stage, and the elimination curves were similar. Elimination was rapid in the liver, kidney, and muscles but slow in the skin and fat.

#### 3.2.3. Elimination Pattern of Lincomycin and Its Main Metabolites

As shown in Figure S3, four hours after drug withdrawal, the residue level of lincomycin reached its peak in the muscles, skin and fat, liver, and kidney. The concentration was the highest in the liver, followed by the kidney, skin and fat, and muscles. However, no related data were obtained in this study. The periods when lincomycin levels dropped below the residue limits stipulated in GB 31650-2019 in the muscles, skin and fat, liver, and kidney were the first, seventh, and second day after drug withdrawal, respectively. Elimination was rapid in the liver, kidney, and muscles but slow in the skin and fat.

#### 3.2.4. Elimination Pattern of Tiamulin and Its Main Metabolites

As shown in Figure S3, four hours after drug withdrawal, the residue level of tiamulin reached its peak in the muscles, skin and fat, liver, and kidney. The concentration was the highest in the skin and fat among the tissues, followed by the kidney, liver, and muscles. The periods when tiamulin levels dropped below the residue limits stipulated in GB 31650-2019 in the muscles, skin and fat, and liver were 4 h after drug withdrawal, the 1st day after drug withdrawal, and 4 h after drug withdrawal, respectively. At present, there is no residue limit set for tiamulin in the kidney. Elimination was rapid in the liver, kidney, and muscles but slow in the skin and fat.

### 3.3. WDTs and MRLs

WDT refers to the interval between the discontinuation of drug administration to animals and the permitted slaughter or the permitted marketing of their products [17].

The maximum residue limits (MRLs) of the seven types of antibiotics in the muscles, skin and fat, liver, and kidney were formulated with reference to GB 31650-2019, as shown in Table 5. Calculated using the WDT calculation software WT1.4, the WDTs of oxytetracycline, chlortetracycline, erythromycin, tylosin, tylvalosin, lincomycin, and tiamulin in Taihang chickens were 3.8 days, 4.6 days, 10.7 days, 7.9 days, 12.5 days, 13 days, and 6.9 days, respectively, as shown in Figure S4. Combined with the data analysis of the eggs analyzed during a pervious study, it is therefore recommended that the WDTs of oxytetracycline, chlortetracycline, erythromycin, tylosin, tiamulin, lincomycin, and tiamulin in Taihang chickens be 4 days, 5 days, 11 days, 8 days, 13 days, 13 days, and 7 days, respectively.

## 4. Discussion

In this study, we aimed to evaluate the WDTs of seven antibiotics in Taihang chickens to ensure the safety of their food products. In a previous study, the egg yolk was identified as the target tissue for estimating the WDTs of the seven investigated drugs. The recommended WDTs of oxytetracycline, chlortetracycline, erythromycin, tylosin, tildipirosin, lincomycin, and tiamulin in Taihang chickens were found to be 3 days, 1 day, 11 days, 3 days, 8 days, 9 days, and 0 days, respectively. We aimed to further enhance the understanding of the drug residues and elimination patterns of the seven antibiotics in Taihang chickens, making the data more complete and the formulation of the WDT more accurate. Based on the results of this study, it was found that the drugs were unevenly distributed in the different bodily tissues. High levels of drug residues were detected in the kidneys and liver in the early stage of drug withdrawal, suggesting that the kidneys and liver are the key tissues of concern for food safety control and toxicology. Oxytetracycline, chlortetracycline, and erythromycin exhibited the highest residue concentrations in the kidneys; tylosin and tildipirosin exhibited the highest residue concentrations in the skin and fat; and lincomycin exhibited the highest residue concentration in the liver. These findings indicate that the main tissues of residue accumulation in chickens vary. The distribution of drugs is generally affected by organ blood flow and tissue affinity. The blood supply to the liver, kidneys, and lungs is higher than that to the muscles and skin and fat [18,19]. Most antibiotics are found in relatively high levels in the liver and kidneys. However, the lipophilic properties of drugs result in higher skin and fat content compared with that in the muscles [20]. From the above results, it is evident that the elimination patterns of antibiotics in different tissues vary, impacting the formulation of the WDT.

The skin is a complex multi-layer tissue. The absorption process of transdermal applied chemicals is complicated by the inherent biological variability of the skin (including factors such as blood flow). The barrier function of the skin is one of its most important characteristics, and its membrane structure is relatively impermeable to aqueous solutions and most ions. The skin of chickens is also covered with a large number of feathers [21]. Given the above factors, the complex structure of the skin may also lead to differences in drug absorption following administration. In this study, macrolides, which are fat-soluble drugs, were found in high residue concentrations in the skin and fat of Taihang chickens, with slow elimination, and the WDT of the skin and fat was longer than that of the liver and kidney tissues. The concentration of water-soluble tetracyclines was relatively much lower but also exhibited slow elimination. However, the results of other related studies [22,23] generally suggest that the WDT of the skin and fat is shorter than that of the liver and kidney tissues and close to that of muscle tissues. One possible explanation for this variation is the fact that during the tissue sampling of the skin and fat in this study, chicken feathers were manually plucked, whereas workers in chicken slaughterhouses employ hot water scalding. The sampling process may have resulted in chicken feathers being present in the final sample, and chicken feathers are considered a matrix with high residue levels in many studies [24,25,26].

The residue limits of the examined antibiotics in the different chicken tissues exhibit variability. Based on the maximum residue limits (MRLs) of the seven antibiotics in the muscles, skin and fat, liver, and kidney stipulated in GB 31650-2019, the WDT was calculated using the WDT calculation software WT1.4. When 40-day-old Ross cocks [27] were orally administered 60 mg/kg chlortetracycline for 5 consecutive days, the elimination rate of chlortetracycline and 4-epi-chlortetracycline in the kidney tissue was much slower than that in the liver and muscle tissues, and the calculated WDT was 3 days. The difference between the above finding and the experimental results of the chlortetracycline group in this study is the fact that elimination in the kidneys in this study was rapid, and the formulated WDT of chlortetracycline was 5 days. When 22-day-old broiler chickens (Ross 308 genetic) were fed 80% tylosin (tylosin tartrate) at a dose of 32 mg/kg body weight for 5 consecutive days, the concentration of tylosin in muscle and liver tissue samples was lower than the detection limit of this method (25 μg/kg and 50 μg/kg) on the 1st day after drug withdrawal [26]. However, in the tylosin group in this study, elimination in tissues was slow. When broiler chickens (Ross 308 genetic) [28] were administered lincomycin at a dose of 50 mg/kg body weight for 7 consecutive days, the residue level in their muscles was 106.41 μg/kg on the 1st day after drug withdrawal and 1294.36 μg/kg in the liver. On the second day after drug withdrawal, the residue level in the muscles was lower than the limit of quantification (73 μg/kg) established using the analytical method, and the liver residue level was lower than 500 μg/kg of the limit of maximum residue (LMRS) (Commission Regulation (EU) 2010). The results were also consistent with the trend seen in this study; however, the residue levels in the various tissues in this study were slightly lower, and the elimination time was marginally slower [28]. When broiler chickens were fed tiamulin via continuous drinking water at a dose of 40 mg/kg body weight for 3 days, it was found that the concentration was the highest in the liver, and no tiamulin residues were detected in the various bodily tissues on the 5th day. The primary difference with this study is the fact that the residue level in the skin and fat in this study was relatively high [29]. When comparing the black-boned chicken with the 817 broiler chicken breed, the tissue residue time was significantly longer, which may be related to the high melanin content in the edible tissues of the black-boned chicken [30]. It is a worthwhile consideration to revise the WDTs based on the residue elimination of antibiotics in specific animal breeds.

The elimination kinetics of the studied drugs in the different tissues vary, and there is no discernible pattern in the elimination rates of the drugs in the different tissues. The elimination rate of the drug in specific tissues should be determined based on the specific circumstances (such as the type of drug, route of administration, and/or animal species, age, and disease status). Factors such as different feeding methods and animal breeds can alter the metabolic elimination cycle of drugs. The impact of the diversity of chicken breeds on the variation in the WDTs of antibiotics poses a great challenge to the establishment of standardized WDTs [31]. In addition, due to differences in the dosage and interval of orally administered drugs, the drug residue concentrations obtained by different researchers also vary [32,33]. The formulation of the WDT is related to factors such as the feeding conditions of animals, their age and breed, and the prescription formulation of drugs. To determine the appropriate WDT of drugs, more in-depth and systematic research is needed. The experimental results presented herein can provide a data basis for the formulation of relevant regulations.

In this study, Taihang chickens were administered drugs via oral feeding, and the content of residue marker substances was detected using the UPLC-MS/MS method. The Committee for Veterinary Medicinal Products of the European Medicines Evaluation Agency recommends determining the WDT when the drug residue concentration is lower than the specified maximum residue limits (MRLs). Based on the residue elimination in each tissue and the MRLs, these values were combined with the previous recommended WDTs of Taihang chickens for oxytetracycline, chlortetracycline, erythromycin, tylosin, tylvalosin, lincomycin and tiamulin, which are 3 days, 1 day, 11 days, 3 days, 8 days, 9 days, and 0 days, respectively. Lastly, the recommended WDTs of Taihang chickens for oxytetracycline, chlortetracycline, erythromycin, tylosin, tylvalosin, lincomycin, and tiamulin are 4 days, 5 days, 11 days, 8 days, 13 days, 13 days, and 7 days, respectively. The Chinese Veterinary Pharmacopoeia (2020 Edition) stipulates that the WDTs for oxytetracycline, chlortetracycline, erythromycin, tylosin, tylvalosin, lincomycin, and tiamulin are 5 days, 7 days, 3 days, 1 day, 5 days, 5 days, and 5 days, respectively. In this study, only the WDTs of oxytetracycline and chlortetracycline for Taihang chickens were found to be shorter than those stipulated in the Chinese Veterinary Pharmacopoeia. The differences between the WDTs of tiamulin for Taihang chickens and those stipulated in the Chinese Veterinary Pharmacopoeia are not significant, and the WDTs of other antibiotics used in Taihang chickens are all longer than those stipulated in the Chinese Veterinary Pharmacopoeia. One possible explanation for this discrepancy is the fact that Taihang chickens rapidly metabolize tetracycline drugs. Regarding the significant prolongation of the WDTs of macrolide drugs and lincomycin, this finding may be related to the relatively slow metabolism of macrolide drugs and lincomycin by Taihang chickens or the delay in the elimination of tissue residues.

## 5. Conclusions

The results presented in this study provide confirmation of the residue elimination times of seven types of antibiotics in the muscles, skin and fat, livers, and kidneys of Taihang chickens following oral administration. The residual concentrations of oxytetracycline, chlortetracycline, and erythromycin are the highest in the kidneys, the residual concentrations of tylosin and tiamulin are the highest in the skin and fat, and the residual concentration of lincomycin is the highest in the liver. Based on residue elimination in each tissue and the maximum residue limits (MRLs), the recommended WDTs for Taihang chickens following the administration of oxytetracycline, chlortetracycline, erythromycin, tylosin, tylvalosin, lincomycin, and tiamulin are 4 days, 5 days, 11 days, 8 days, 13 days, 13 days, and 7 days, respectively. In future studies, it is recommended to combine pharmacokinetic data to further verify the scientific merit of the specific WDTs for Taihang chickens and promote the alignment of the WDTs for characteristic breeds with the standards of the Chinese Veterinary Pharmacopoeia.

## Figures and Tables

**Table 1:** Antibiotics, manufacturers, and batch numbers used in this study.

Drug Name	Chinese Veterinary Pharmacopoeia WDT	Manufacturer and Batch Number
50% oxytetracycline hydrochloride soluble powder	5 days	Hebei Xiangda Hezhong Biotechnology Co., Ltd. (Shijiazhuang, China) batch number 20220702
20% chlortetracycline hydrochloride soluble powder	7 days	Baoding Ji Zhong Pharmaceutical Co., Ltd. (Baoding, China) batch number 20220703
5% erythromycin thiocyanate soluble powder	3 days	Baoding Ji Zhong Pharmaceutical Co., Ltd. batch number 20220906
50% tylosin tartrate soluble powder	1 days	Baoding Ji Zhong Pharmaceutical Co., Ltd. batch number 202202002
20% tylvalosin tartrate soluble powder	5 days	Zhangjiakou Wanquan District Ketai Biotechnology Co., Ltd. (Zhangjiakou, China) batch number 22081401
5% lincomycin hydrochloride soluble powder	5 days	Hebei Zhenghe Biopharmaceutical Co., Ltd. (Xingtai, China) batch number 20230043
45% tiamulin fumarate soluble powder	5 days	Hebei Weili Biotechnology Co., Ltd. (Shijiazhuang, China) batch number 20220538

**Table 2:** Detection conditions for the seven types of antibiotics.

Analyte	Retention Time/min	Precursor Ion (*m*/*z*)	Daughter Ion (*m*/*z*)	Collision Voltage/V	Cone Voltage/V
Oxytetracycline	2.50	460.8	200.7	44	2
			425.8	20	
Chlortetracycline	3.28	478.8	153.7	26	34
			443.8	20	
Erythromycin A	3.45	734.2	158.1	30	30
			576.2	18	
Tylosin A	3.47	916.2	145.1	35	20
			174.1	35	
Tylvalosin	3.76	1042.7	109.1	45	30
			174.1	45	
3-O-Acetyltylosin	3.56	958.5	174.1	44	30
			772.4	44	
Lincomycin	2.04	407.0	126.2	22	30
			359.2	16	
Tiamulin	3.59	494.4	119.0	25	10
			192.1	19	

**Table 3:** Methodological parameters.

Residual Marker	Regression Equation	R^2^ Correlation Coefficient	LOD/(µg/kg)	LOQ/(µg/kg)
Oxytetracycline	y = 13037.3x + 11524.2	0.9977	0.5	2.0
Chlortetracycline	y = 8021.96x − 964.676	0.9995	0.5	2.0
Erythromycin A	y = 2682.41x − 1897.21	0.9958	0.5	2.0
Tylosin A	y = 7941.16x + 14,196	0.9913	1.0	5.0
Tylvalosin	y = 180.583x + 68.504	0.9987	0.2	1.0
3-O-Acetyltylosin	y = 3130.63x + 6298.73	0.9919	1.0	5.0
Lincomycin	y = 1835.12x + 3640.36	0.9959	0.2	1.0
Tiamulin	y = 92568.2x + 162944	0.9951	0.2	1.0

**Table 4:** The accuracy, precision, CCα, and CCβ of the method for the determination of the seven types of antibiotics in muscle, skin and fat, liver, and kidney samples.

Analyte	Matrix	Spiked Concentration (μg/kg)	Recovery (%) (*n* = 6)	Intra-Day RSD (%) (*n* = 6)	Inter-Day RSD (%) (*n* = 6)	CCα (μg/kg)	CCβ (μg/kg)
Oxytetracycline	Muscle	10	76	3.7	4.4	0.322	0.424
	Skin and fat	10	74	3.6	3.9	0.316	0.342
	Liver	10	72	3.9	3.0	0.327	0.502
	Kidney	10	73	4.1	3.8	0.388	0.446
	Muscle	25	71	3.8	4.4	0.302	0.507
	Skin and fat	25	73	3.7	3.8	0.296	0.469
	Liver	25	72	4.0	2.9	0.309	0.478
	Kidney	25	74	3.5	3.7	0.326	0.338
	Muscle	50	73	3.4	3.6	0.296	0.471
	Skin and fat	50	72	3.9	3.7	0.283	0.482
	Liver	50	75	3.1	4.1	0.277	0.422
	Kidney	50	73	4.0	3.9	0.293	0.433
Chlortetracycline	Muscle	10	72	3.8	2.8	0.269	0.463
	Skin and fat	10	73	4.1	3.2	0.299	0.452
	Liver	10	72	3.7	3.7	0.324	0.437
	Kidney	10	71	4.0	2.0	0.304	0.439
	Muscle	25	74	3.3	2.8	0.253	0.476
	Skin and fat	25	73	3.8	3.1	0.266	0.442
	Liver	25	72	3.6	2.5	0.284	0.471
	Kidney	25	73	2.9	2.7	0.256	0.455
	Muscle	50	73	3.2	3.4	0.278	0.493
	Skin and fat	50	72	2.7	3.3	0.301	0.402
	Liver	50	71	2.5	2.6	0.261	0.443
	Kidney	50	74	4.0	2.3	0.263	0.461
Erythromycin A	Muscle	10	80	2.4	3.4	0.343	0.458
	Skin and fat	10	78	2.5	3.2	0.326	0.404
	Liver	10	80	1.9	3.6	0.335	0.426
	Kidney	10	82	2.6	3.7	0.336	0.472
	Muscle	25	77	3.0	3.8	0.338	0.476
	Skin and fat	25	80	2.9	3.1	0.345	0.435
	Liver	25	79	2.3	3.3	0.321	0.472
	Kidney	25	78	2.7	3.5	0.348	0.451
	Muscle	50	81	2.8	3.4	0.320	0.445
	Skin and fat	50	80	2.2	3.2	0.329	0.423
	Liver	50	78	3.1	2.9	0.335	0.444
	Kidney	50	79	2.5	3.1	0.343	0.458
Tylosin A	Muscle	10	72	2.8	3.7	0.336	0.426
	Skin and fat	10	71	2.7	2.4	0.272	0.415
	Liver	10	70	3.6	2.9	0.256	0.416
	Kidney	10	73	4.2	2.4	0.339	0.457
	Muscle	25	72	2.6	3.2	0.248	0.452
	Skin and fat	25	72	3.4	2.6	0.337	0.437
	Liver	25	72	3.2	2.7	0.323	0.462
	Kidney	25	71	2.7	2.3	0.293	0.474
	Muscle	50	73	3.2	3.1	0.268	0.433
	Skin and fat	50	70	2.8	2.8	0.262	0.402
	Liver	50	72	3.6	2.2	0.274	0.413
	Kidney	50	71	2.5	3.5	0.291	0.472
Tylvalosin	Muscle	10	83	2.4	3.7	0.293	0.347
	Skin and fat	10	80	3.4	3.6	0.296	0.373
	Liver	10	81	2.9	3.8	0.285	0.362
	Kidney	10	82	3.5	4.3	0.320	0.415
	Muscle	25	81	3.2	4.0	0.334	0.417
	Skin and fat	25	83	2.7	2.9	0.326	0.422
	Liver	25	82	2.2	3.3	0.313	0.408
	Kidney	25	80	3.0	4.0	0.306	0.411
	Muscle	50	84	3.3	3.8	0.315	0.433
	Skin and fat	50	81	3.1	3.6	0.318	0.418
	Liver	50	84	3.6	3.5	0.316	0.454
	Kidney	50	83	2.8	3.2	0.308	0.437
3-O-Acetyltylosin	Muscle	10	75	3.2	3.3	0.295	0.394
	Skin and fat	10	74	3.5	3.2	0.310	0.403
	Liver	10	73	3.6	2.9	0.325	0.415
	Kidney	10	72	3.4	3.0	0.333	0.428
	Muscle	25	73	3.7	2.7	0.325	0.444
	Skin and fat	25	74	3.2	2.8	0.316	0.438
	Liver	25	75	3.3	3.1	0.286	0.466
	Kidney	25	76	2.9	3.5	0.347	0.454
	Muscle	50	72	2.8	3.6	0.322	0.461
	Skin and fat	50	73	3.0	2.8	0.358	0.463
	Liver	50	74	2.8	3.1	0.336	0.428
	Kidney	50	75	3.2	2.7	0.325	0.409
Lincomycin	Muscle	10	92	2.8	2.9	0.302	0.422
	Skin and fat	10	90	3.1	2.8	0.254	0.418
	Liver	10	91	2.9	2.4	0.337	0.434
	Kidney	10	90	3.3	2.8	0.303	0.416
	Muscle	25	88	2.7	3.2	0.349	0.423
	Skin and fat	25	92	2.6	2.5	0.382	0.447
	Liver	25	89	2.3	2.8	0.301	0.417
	Kidney	25	91	2.5	3.2	0.267	0.426
	Muscle	50	93	2.4	2.9	0.283	0.427
	Skin and fat	50	90	2.4	2.7	0.277	0.429
	Liver	50	93	3.1	2.2	0.269	0.415
	Kidney	50	94	2.7	2.7	0.284	0.433
Tiamulin	Muscle	10	99	3.6	2.1	0.245	0.472
	Skin and fat	10	98	3.8	2.3	0.223	0.401
	Liver	10	97	3.1	2.0	0.265	0.454
	Kidney	10	98	3.0	2.2	0.222	0.428
	Muscle	25	99	3.0	1.7	0.257	0.481
	Skin and fat	25	97	2.8	2.4	0.241	0.477
	Liver	25	95	3.3	2.7	0.270	0.468
	Kidney	25	98	3.2	3.4	0.259	0.426
	Muscle	50	96	2.9	1.9	0.266	0.461
	Skin and fat	50	97	2.6	2.1	0.238	0.503
	Liver	50	99	2.2	1.8	0.274	0.432
	Kidney	50	96	3.3	1.9	0.233	0.486

**Table 5:** GB 31650-2019 MRLs and WDTs.

Veterinary Drug	Sample	31650 MRLs	CAC MRLs	EU MRLs	Chinese Veterinary Pharmacopoeia WDT	Recommended WDT in Taihang Chickens
Oxytetracycline	muscle	200	200	200	5 days	4 days
	liver	600	600	600		
	kidney	1200	1200	1200		
Chlortetracycline	muscle	200	200	200	7 days	5 days
	liver	600	600	600		
	kidney	1200	1200	1200		
Erythromycin	muscle	100	100	100	3 days	11 days
	skin and fat	100	100	100		
	liver	100	100	100		
	kidney	100	100	100		
Tylosin	muscle	100	100	100	1 days	8 days
	skin and fat	100	100	100		
	liver	100	100	100		
	kidney	100	100	100		
Tylvalosin	skin and fat	50		50	5 days	13 days
	liver	50		50		
Lincomycin	muscle	200	200	200	5 days	13 days
	skin and fat	100	100	100		
	liver	500	500	500		
	kidney	500	500	500		
Tiamulin	muscle	100		100	5 days	7 days
	skin and fat	100		100		
	liver	1000		1000		

## Data Availability

All of the datasets collected and analyzed during the current study are available from the corresponding author upon request; the availability of the data is restricted to investigators based at academic institutions.

## Supplementary Materials

The following supporting information can be downloaded at: https://www.mdpi.com/article/10.3390/ani15152219/s1. Figure S1: Residue depletion curves of Oxytetracycline (A) and Chlortetracycline (B) in muscle, skin+fat, liver and kidney. Figure S2: Residue depletion curves of Erythromycin A (A), Tylosin A (B) and Tylvalosin (C) in muscle, skin+fat, liver and kidney. Figure S3: Residue depletion curves of Lincomycin (A) and Tiamulin (B) in muscle, skin+fat, liver and kidney. Figure S4: Calculation of the withdrawal times for oxytetracycline, chlortetracycline, erythromycin, tylosin, tylvalosin, lincomycin, and tiamulin to ensure that they were kept below the MRLs (with 95% tolerance limits and 95% confidence intervals). Each circle represents the individual concentration of drug measured per day.
