# Supplementary material for: Residue Elimination Patterns and Determination of the Withdrawal Times of Seven Antibiotics in Taihang Chickens
Source: Animals (Basel). 2025 Jul 28;15(15):2219. doi: 10.3390/ani15152219 (PMC12345420; doi:10.3390/ani15152219)
Supplement: Supplementary file 1 [file animals-15-02219-s001.zip › animals-3745070-supplementary.pdf]

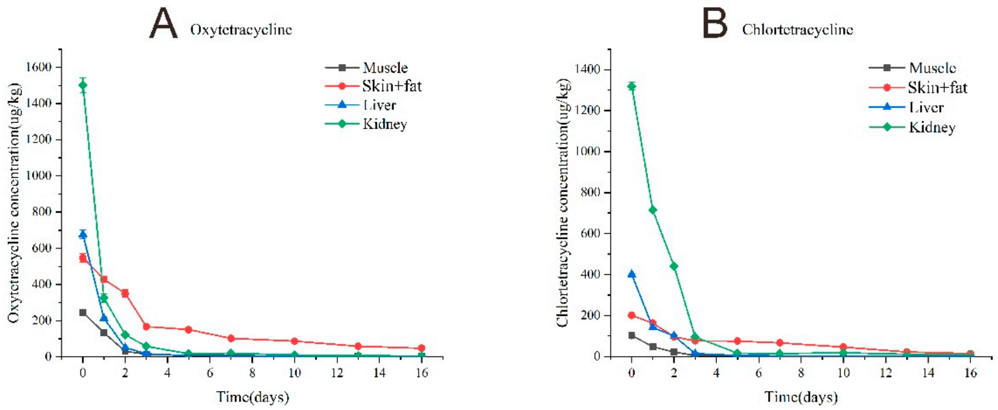

Figure S1：Residue depletion curves of Oxytetracycline（A）and Chlortetracycline（B）in muscle, skin+fat, liver and kidney.

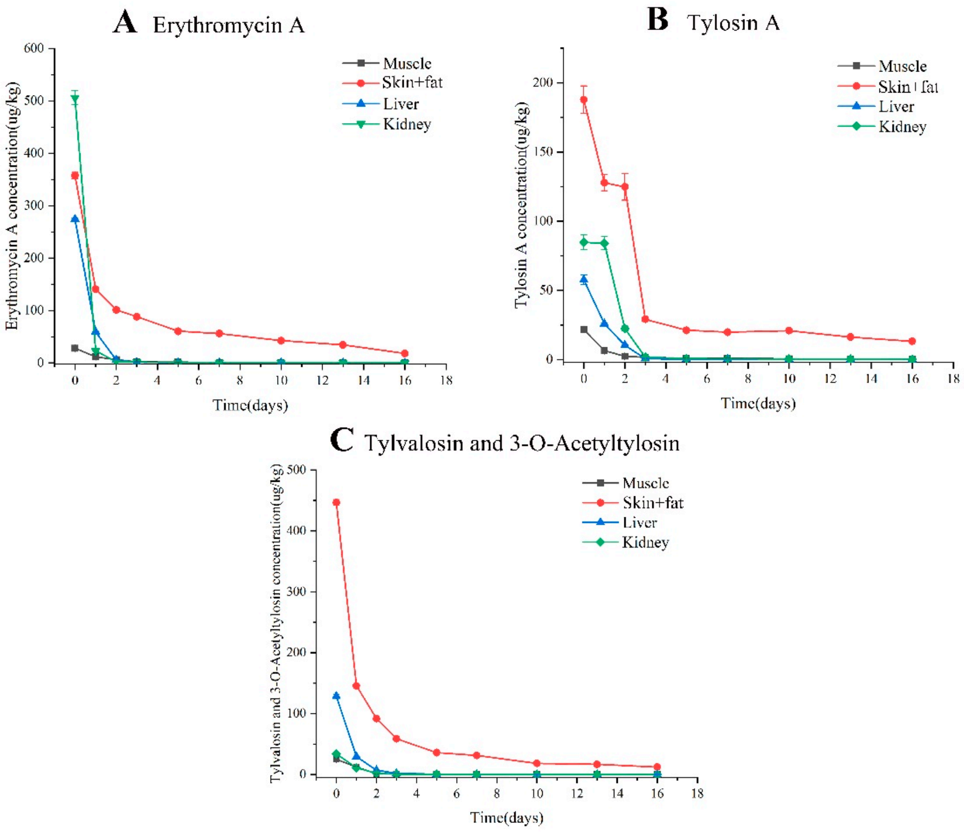

Figure S2：Residue depletion curves of Erythromycin A(A)、Tylosin A(B) and Tylvalosin(C) in muscle, skin+fat, liver and kidney.

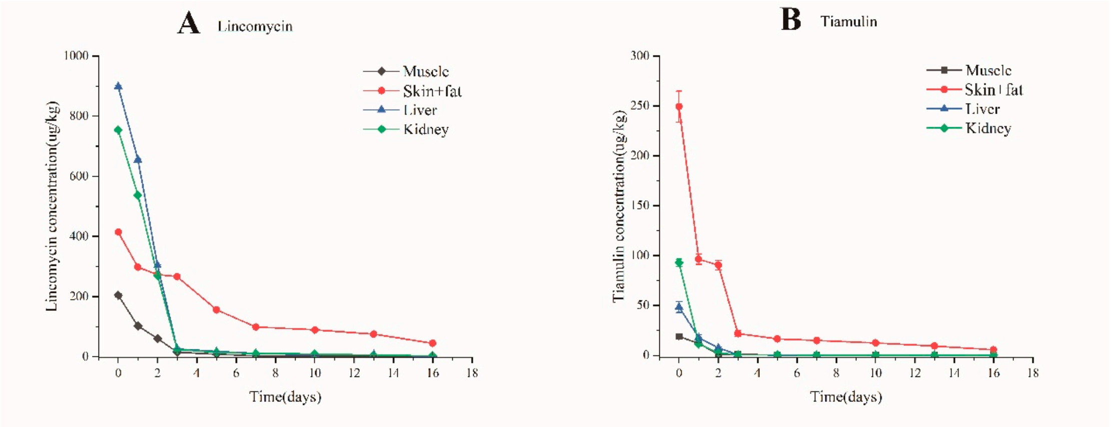

Figure S3: Residue depletion curves of Lincomycin（A）and Tiamulin（B）in muscle, skin+fat, liver and kidney.

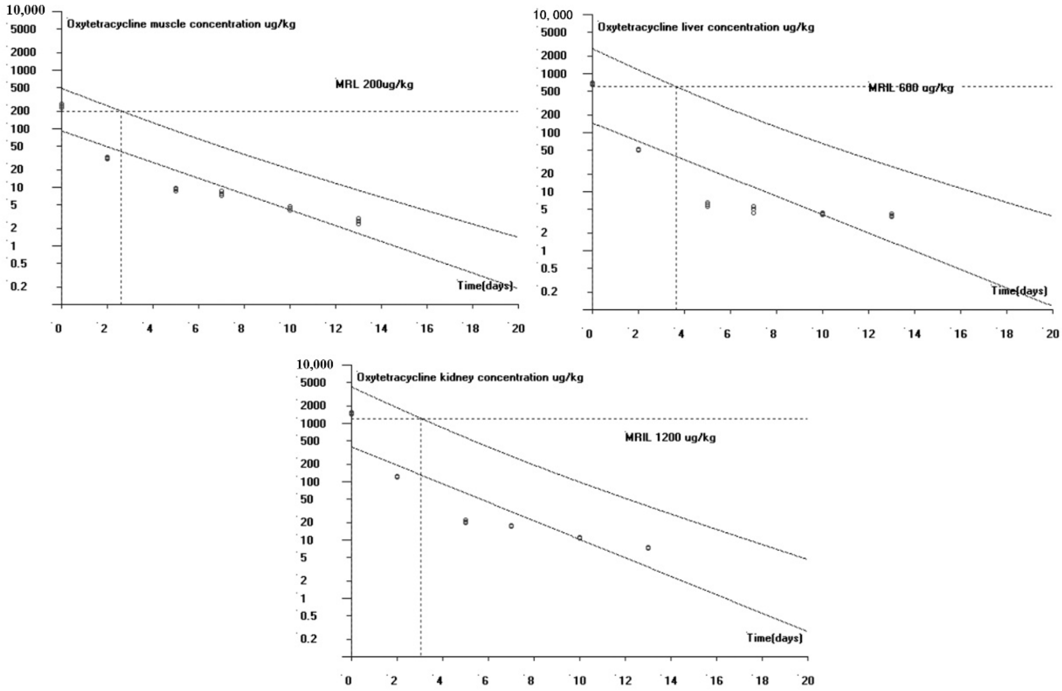

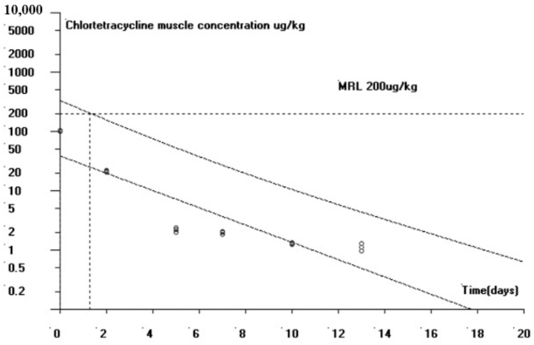
Chlortetracycline muscle concentration ug/kg
MRL 200ug/kg
Time(days)

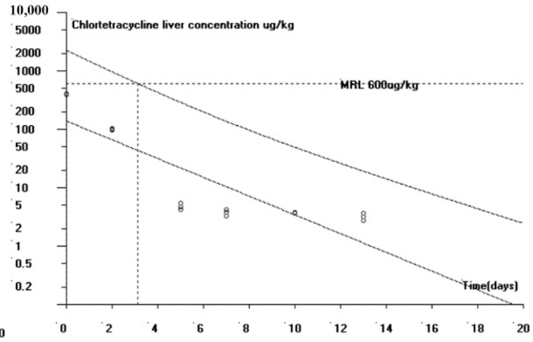
Chlortetracycline liver concentration ug/kg
MRL 600ug/kg
Time(days)

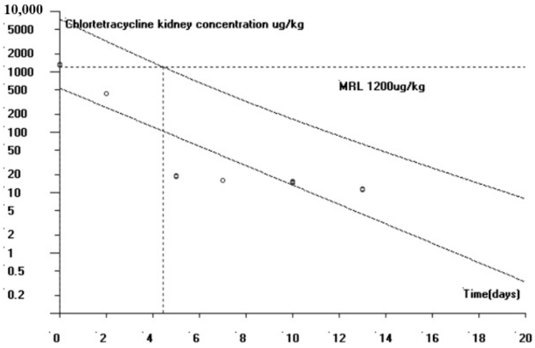
Chlortetracycline kidney concentration ug/kg
MRL 1200ug/kg
Time(days)

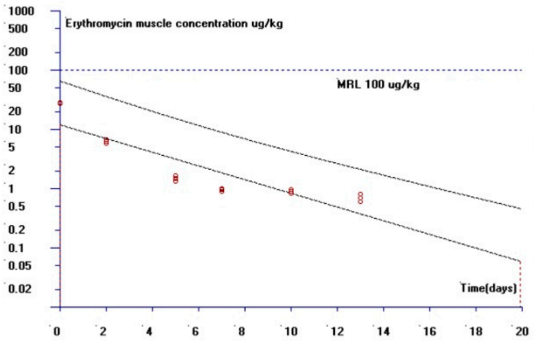
Erythromycin muscle concentration ug/kg
MRL 100 ug/kg
Time(days)

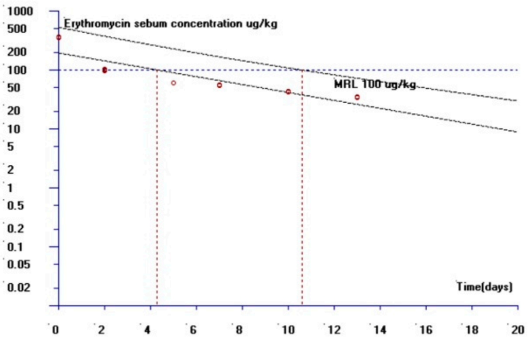
Erythromycin sebum concentration ug/kg
MRL 100 ug/kg
Time(days)

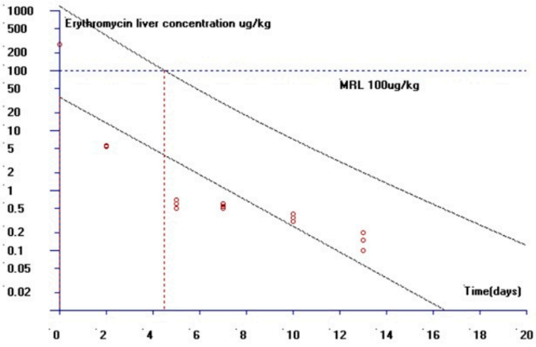
Erythromycin liver concentration ug/kg
MRL 100ug/kg
Time(days)

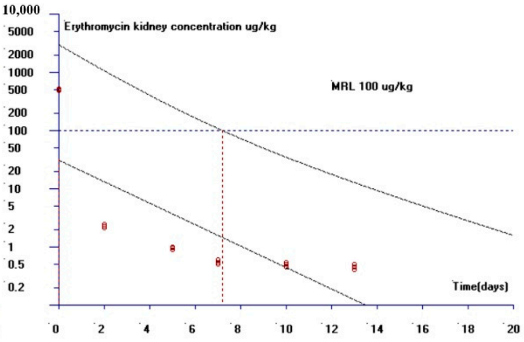
Erythromycin kidney concentration ug/kg
MRL 100 ug/kg
Time(days)

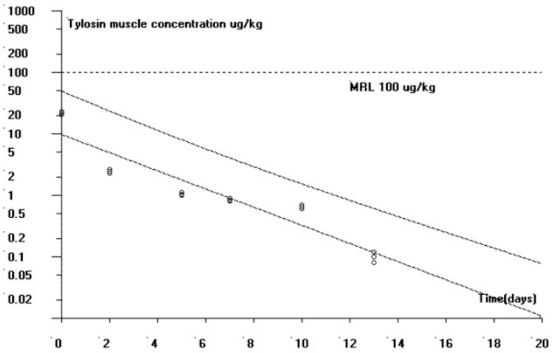
Tylosin muscle concentration ug/kg
MRL 100 ug/kg
Time(days)

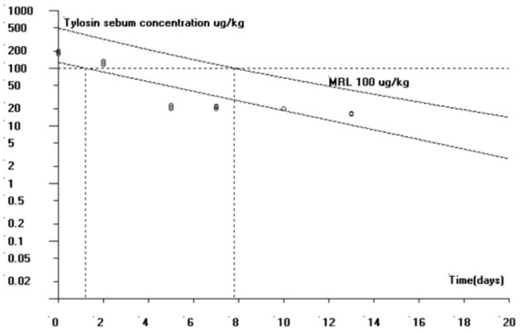
Tylosin sebum concentration ug/kg
MRL 100 ug/kg
Time(days)

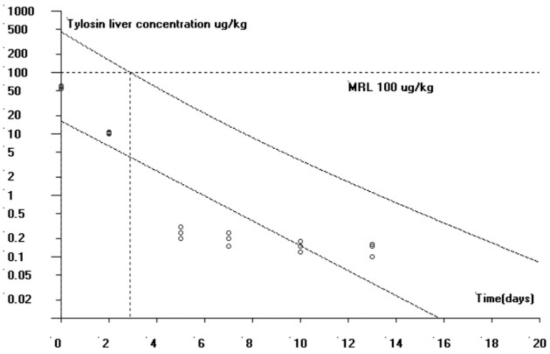
Tylosin liver concentration ug/kg
MRL 100 ug/kg
Time(days)

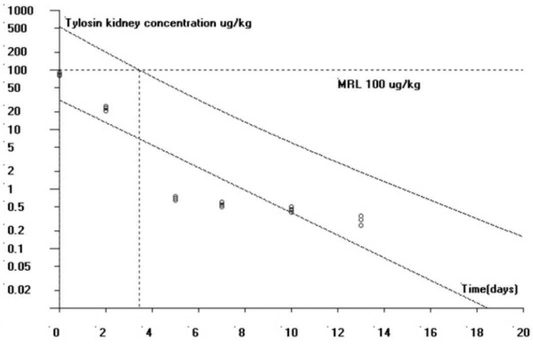
Tylosin kidney concentration ug/kg
MRL 100 ug/kg
Time(days)

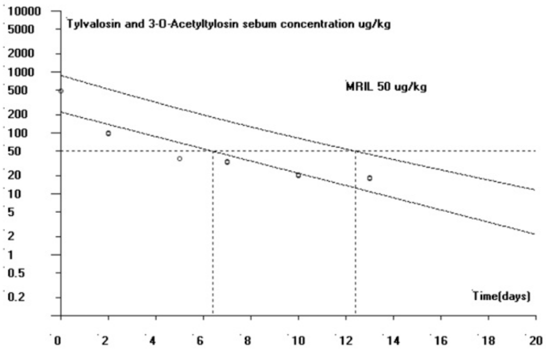
Tylvalosin and 3-O-Acetyltylosin sebum concentration ug/kg
MRIL 50 ug/kg
Time(days)

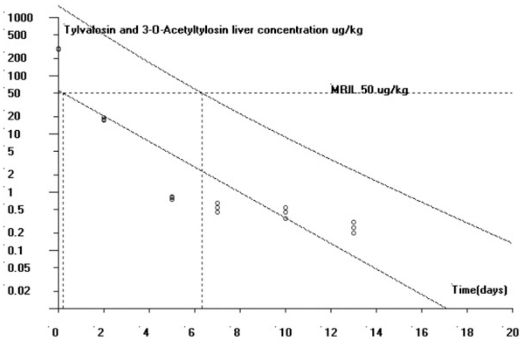
Tylvalosin and 3-O-Acetyltylosin liver concentration ug/kg
MRIL 50 ug/kg
Time(days)

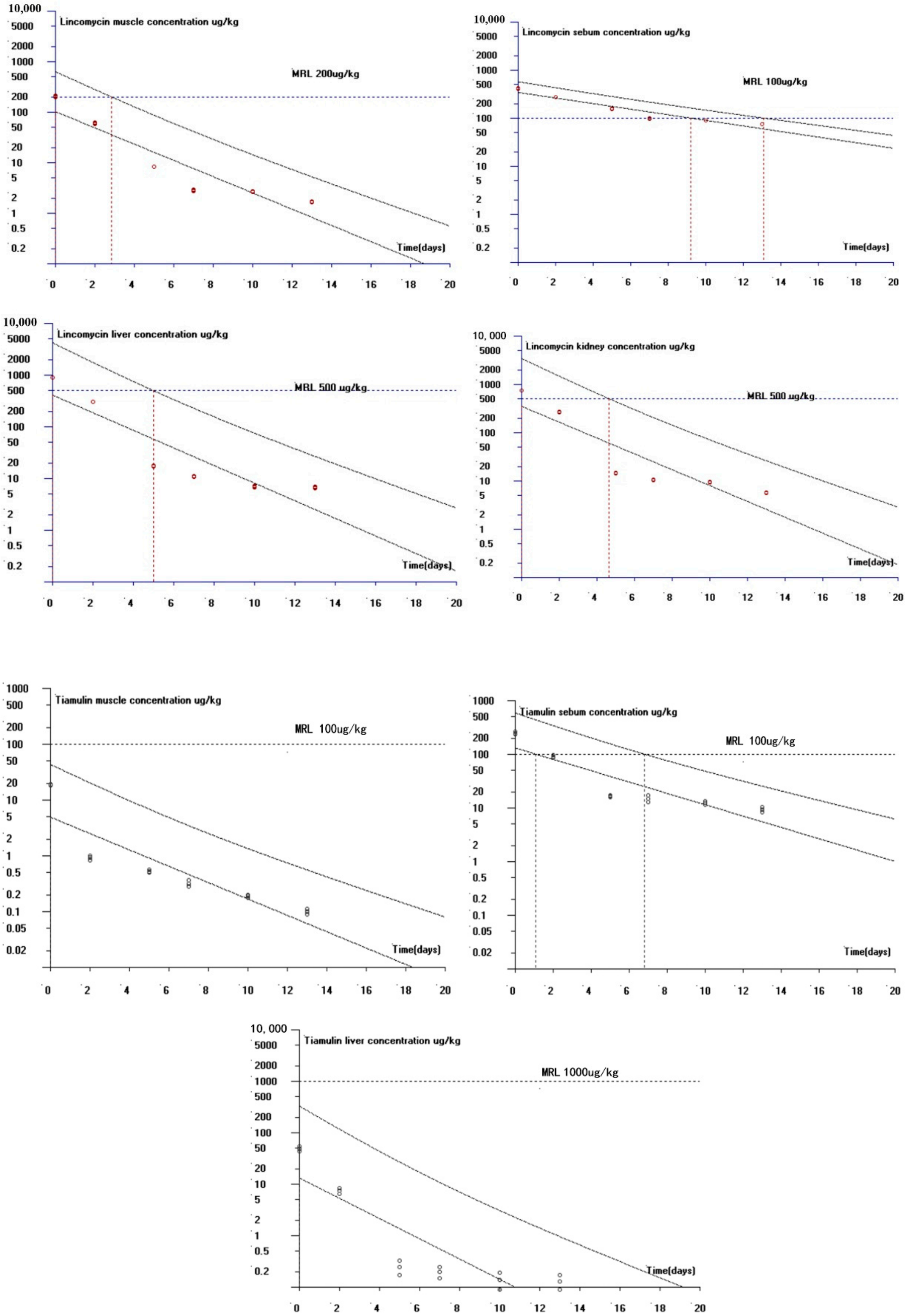

Figure S4: Calculation of the withdrawal times for oxytetracycline, chlortetracycline, erythromycin, tylosin, tylvalosin, lincomycin, and tiamulin to ensure that they were kept below the MRLs (with 95% tolerance limits and 95% confidence intervals). Each circle represents the individual concentration of drug measured per day.
